# Effect of Different Regions and Ensiling Periods on Fermentation Quality and the Bacterial Community of Whole-Plant Maize Silage

**DOI:** 10.3389/fmicb.2021.743695

**Published:** 2021-11-10

**Authors:** Yuan Huang, Longfei Liang, Sheng Dai, Changrong Wu, Chao Chen, Jun Hao

**Affiliations:** Department of Grassland Science, College of Animal Science, Guizhou University, Guiyang, China

**Keywords:** whole-plant maize, silage, fermentation quality, microbial community, environmental factor

## Abstract

This study aimed to explore the changes in the microbial community on the silage material surface and during the ensiling process of whole-plant maize in different regions. Whole-plant maize silages were sampled in Ziyun, Guanling, and Weinning counties within warm and humid climate areas in southern China. Silages were sampled at 0, 2, 5, 10, 20, and 45 days during ensiling. The nutritional components, fermentation properties, and microbiomes were examined to evaluate the influence of sampling area and fermentation time on the quality of silage. The results showed that the pH values of all silages significantly decreased (<4.2 at ensiling day 2) during fermentation and all silages achieved satisfactory fermentation at 45 days. Butyric acid was not detected during ensiling, and the contents of acetic acid and ammonia nitrogen in the final silages were below 6 g/kg DM and 50 g/kg total nitrogen, respectively. *Weissella* was the dominant epiphytic bacteria of raw material in Ziyun and Weinning, while *Lactobacillus* was prevalent in Guanling. *Lactobacillus* dominated the ensiling process, and its abundance significantly increased with increasing fermentation time in the three groups. *Lactobacillus* was negatively correlated with pH of all silages (*p* < 0.05) and positively correlated with lactic acid, propionic acid and acetic acid (*p* < 0.05). Furthermore, the bacterial community was significantly correlated with environmental factors. Altitude had a highly positive correlation with the abundance of *Stenotrophomonas*, *Chryseobacterium*, and *Massilia* (*p* < 0.01), while precipitation was negatively correlated with these bacteria. The humidity and average temperature significantly influenced the *Lactobacillus* and *Weissella* abundances of fresh whole-plant maize. During the ensiling process, the silages from three regions had similar bacterial dynamic changes, and the *Lactobacillus* formed and maintained good fermentation characteristics in whole-plant maize silage.

## Introduction

Ensiling is a common method for preserving fresh forage and contributes to an uninterrupted supply of forage feedstuff to ruminant animals (Pahlow et al., 2003). Lactic acid bacteria (LAB) are the most important beneficial bacteria in the process of ensiling fermentation (Zhu et al., 2010), which results in organic acids with water-soluble carbohydrate (WSC) as fermentation substrate under anaerobic conditions to reduce pH value for achieving the purpose of long-term preservation of silage (Keshri et al., 2018). It is well established that LAB regularly relate to silage fermentation belong to the genera *Enterococcus*, *Lactobacillus, Lactococcus, Leuconostoc, Pediococcus, Streptococcus*, and *Weissella* (Pang et al., 2011; Ni et al., 2018; Yang et al., 2019). The quality of silage mainly depends on the composition and abundance of microbial communities during ensiling (Kung et al., 2018; Zi et al., 2021a). Whole-plant maize silage has a better absorption rate and higher nutritional value than other silages, and is the most common feed for ruminants worldwide (Khan et al., 2015; Zhang et al., 2019). Various silage microbial compositions have been found in different forages (Guo et al., 2018; Xu et al., 2019; Yuan et al., 2019). In addition, for maize ensiling, previous studies have reported that *Lactobacillus* was the dominant contributors during ensiling and subsequent exposure to air (Lin et al., 1992; Zhou et al., 2016; Hu et al., 2018). Generally, the composition of microorganisms changes greatly before and after ensiling (Sepehri and Sarrafzadeh, 2019). Therefore, it is of great significance to further study the microbial diversity changes in silage for understanding the whole fermentation process and finding the root cause of fermentation quality changes.

As previously mentioned, uncontrollable climatic conditions affect silage fermentation, microorganisms and aerobic stability (Bernardes et al., 2018; Guan et al., 2018). Ensiling at high temperatures reduces both the lactic acid (LA) concentration and aerobic stability (Ashbell et al., 2002). Moreover, it has been reported that temperature affects the bacterial diversity and fermentation quality of silage (Wang et al., 2019; Li et al., 2021). Epiphytic bacterial communities in fresh forage depends mainly on geographical location, climate, and cutting (Guo et al., 2018; Yang et al., 2019). Cai et al. (1999) studied the microorganisms attached to the surface of corn, sorghum, alfalfa and Italian ryegrass collected from the same place and found that there were very few *Pediococcus* attached to the surface of sorghum and ryegrass and few *Lactobacillus* attached to the surface of alfalfa. The community of epiphytic bacteria in corn material has been shown to be affected by rainfall and humidity, and the microbial community during ensiling has been shown to be affected by temperature (Guan et al., 2018).

The composition of the epiphytic community is an important factor of silage quality and changes in the microbial community during fermentation (Gharechahi et al., 2017). Nevertheless, the effect of the environment on the epiphytic community of whole-plant maize silage has rarely been reported. We hypothesized that the succession characteristics of whole-plant maize silage are different in different areas. Therefore, this study aimed to evaluate the correlation between bacterial communities and sampling area environmental factors (altitude, precipitation, temperature, and humidity) and then to examine the dynamic changes in microbial community diversity and fermentation quality during the ensiling of whole-plant maize.

## Materials and Methods

### Study Sites and Sample Collection

**FIGURE 1:**
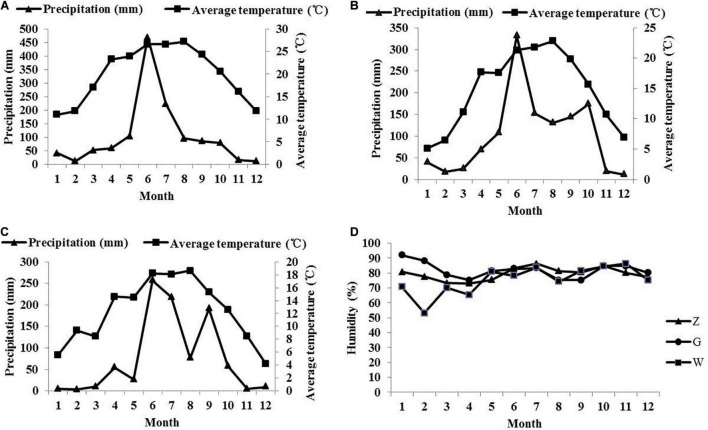
The monthly changes of temperature and precipitation in the area of Ziyun **(A),** Guanling **(B),** and Weining **(C)** in 2019, and monthly humidity changes in three regions **(D)**.

Whole-plant maize was planted in Ziyun County (Z) (106°10′E, 25°37′N, altitude 840 m), Guanling County (G) (105°24′E, 25°57′N, altitude 1350 m), and Weining County (W) (104°16′E, 26°55′N, altitude 2230 m). The monthly changes of temperature, precipitation and humidity in the three regions in 2019 are shown in Figure 1. The variety of maize was Qingfeng No. 4. The samples were planted on February 25, 2019; March 28, 2019; and April 2, 2019 in Ziyun, Guanling, and Weining, respectively. Samples were collected at the dough stage according to local tradition on July 31, 2019; September 12, 2019; and September 18, 2019. Instantly, the materials were collected and transported in ice boxes and stored at –80°C before use. Another part of the collected materials was chopped to approximately 2 cm by a hand paper cutter. After mixing thoroughly, the material was vacuum-packed into plastic bags. Each bag contained approximately 500 g of fresh matter. A total of 54 bags (three treatments × six ensiling durations × three replicates) were prepared and stored at normal temperature (25–30°C), and the fermentation time of all samples was approximately 45 days. Samples were used to determine the chemical composition, fermentation quality and microbial community on days 0, 2, 5, 10, 20, and 45.

### Chemical and Fermentation Profile Analyses

Specimens were determined by drying the samples in a forced air oven at 65°C for 72 h and passed through a 1.0 mm sieve before the chemical assay. The contents of dry matter (DM) and crude protein (CP) were measured according to previously published study (AOAC, 2000). The WSC content was determined by colorimetric after-reaction with anthrone reagent (Turula et al., 2010). The neutral detergent fiber (NDF) and acid detergent fiber (ADF) were analyzed using a previously established method (Van Soest et al., 1991). To measure fermentation quality, 20 g samples were suspended in 180 mL of distilled water overnight at 4°C and filtered through four layers of cheesecloth. Then, the filtrates were used to determine the pH value and ammonia-N (NH_3_-N) and organic acid contents. The pH, lactic acid (LA), acetic acid (AA), propionic acid (PA), butyric acid (BA), ammonia-N (AN), and total nitrogen (TN) concentrations were measured as previously established (Li M. et al., 2019).

### Bacterial Community Analysis

The DNA extraction was operated using the HiPure Soil DNA extraction kit (Magen, Guangzhou, China) according to the manufacturer’s instructions. The 16S rDNA V5-V7 region of the ribosomal RNA gene were amplified by PCR (94°C for 2 min, followed by 30 cycles at 98°C for 10 s, 62°C for 30 s, and 68°C for 30 s and a final extension at 68°C for 5 min) with primers 799F (AACMGGATTAGATACCCKG) and 1193R (ACGTCATCCCCACCTTCC) (Logue et al., 2016). Amplicons were extracted from 2% agarose gels and purified using the AxyPrep DNA Gel Extraction Kit (Axygen Biosciences, Union City, CA, United States) according to the manufacturer’s instructions and quantified using ABI StepOnePlus Real-Time PCR System (Life Technologies, Foster City, United States). Purified amplicons were pooled in equimolar and paired-end sequenced (2 × 250) on an Illumina platform according to the standard protocols.

Tag assembly was carried out using filtered reads according to the following principles: the overlap between paired-end reads should be more than 10-bp and have less than a 2% mismatch. The unique tags were obtained by removal of redundant tags using MOTHUR software (Schloss et al., 2009). The effective tags were clustered into operational taxonomic units (OTUs) of ≥97% similarity using UPARSE (version 9.2.64) pipeline. Diversity metrics were determined using the core-diversity plug-in within QIIME (Callahan et al., 2016). The microbial diversity within an individual sample was assessed using the following alpha diversity indices: the Chao1 richness estimator and Shannon diversity index. Beta diversity was analyzed to assess the structural variation of microbiota across specimens, and then principal component analysis (PCA) was conducted (Vázquez-Baeza et al., 2013). The relative abundances of different bacterial communities at the phylum and genus levels were also analyzed. Pearson correlation coefficient between environmental factors and species was calculated in R project psych package (version 1.8.4). The heat map function of the R software and genus information for the Whole-plant silages were used to generate a heat map. Environmental factors during corn growth and fermentation quality after silage were selected for Spearman correlation analysis with bacterial community. The data were analyzed using the free online platform of Omicshare tools.

### Statistical Analyses

The statistical analyses of two-way ANOVA were performed using SPSS 20.0. Duncan’s HSD test was employed to determine the differences in the treatment means, where significant differences were declared at *P* < 0.05, and the data are expressed as the mean and the standard error of the mean (SEM).

## Results

### Chemical Analysis of Whole-Plant Maize Ensiling

The changes in nutritional components during different regions of ensiling are shown in Table 1. The DM contents of all maize raw materials ranged from 268.6 to 303.3 g/kg, while the highest DM content was 303.3 g/kg in the W group (*P* < 0.05), and the lowest DM content was 268.6 g/kg in the G group (*P* < 0.05). The content of DM in each group decreased with increasing fermentation time and was in the order of W > Z > G samples at any stage of fermentation. The content of CP in the Z and G groups was lower than that in the W group on days 5 and 10 (*P* < 0.05), while that in the Z group was lower than that in the G and W groups on day 45 (*P* < 0.05). The WSC content of each group decreased significantly throughout the fermentation process. The contents of NDF and ADF in the Z group were significantly higher than those in the other groups at any stage of fermentation, and they showed a similar decreasing trend with the extension of fermentation time. Moreover, the contents of NDF and ADF in the G group were lower than those in the other groups on day 45 (*P* < 0.05). The ensiling time (D) and treatment (T) significantly affected the chemical composition (*P* < 0.001). The results also showed a significant interaction between D and T for the contents of DM, WSC, NDF, and ADF (*P* < 0.001).

**TABLE 1:** Chemical composition of whole-plant maize silage in different regions.

**Item**	**Treatment**	**Ensiling days**						**SEM**	***P*-value**		
		**0**	**2**	**5**	**10**	**20**	**45**		**D**	**T**	**D × T**
Dry matter (g/kg FW)	Z	274.1Ba	268.6Bb	262.0Bc	254.9Bd	254.4Bd	250.9Be	0.36	***	***	***
	G	268.6Ca	261.7Cb	254.5Cc	252.6Cd	250.7Ce	248.6Cf				
	W	303.3Aa	293.6Ab	292.1Ac	274.8Ad	264.7Ae	260.6Af				
Water-soluble carbohydrate (g/kg DM)	Z	86.9Aa	66.0Ab	59.0Ac	42.6Ad	32.9Ae	25.2Af	0.42	***	***	***
	G	78.9Ba	58.4Bb	48.7Bc	40.1Bd	33.3Ae	22.6Bf				
	W	72.0Ca	51.4Cb	39.7Cc	35.4Cd	31.5Be	21.3Bf				
Crude protein (g/kg DM)	Z	74.3Cb	71.3Bb	74.0Cb	83.1Ba	72.9Cb	72.5Cb	0.74	***	***	***
	G	84.5Aa	81.8Ab	78.5Bc	76.6Cd	79.1Bc	78.7Ac				
	W	78.4Bc	73.7Bd	83.0Ab	90.1Aa	89.0Aa	74.2Bd				
Starch (g/kg DM)	Z	267.9Ca	262.0Ab	259.4Bc	258.7Bc	255.6Bd	240.8Be	1.17	***	***	***
	G	275.0Ba	252.0Bbc	252.9Cb	250.6Ccd	248.7Cd	244.1Be				
	W	296.2Aa	260.8Ac	272.4Ab	275.3Ab	276.1Ab	275.1Ab				
Neutral detergent fiber (g/kg DM)	Z	527.1Aa	525.7Aa	522.4Aa	515.8Ab	511.0Ab	475.0Ac	1.73	***	***	***
	G	504.1Ba	496.5Bb	495.0Bb	488.5Bc	476.0Bd	456.2Be				
	W	477.3Ca	461.5Cb	457.8Cb	444.0Cc	439.7Cd	437.1Cd				
Acid detergent fiber (g/kg DM)	Z	257.9Aa	255.4Ab	252.9Ac	250.2Ad	248.6Ae	246.9Af	0.64	***	***	***
	G	250.7Ba	244.3Bb	236.4Bc	226.6Cd	222.4Ce	207.3Cf				
	W	244.3Ca	237.4Cb	232.4Cc	229.5Bd	227.4Be	225.4Bf				

Z, Ziyun; G, Guanling; W, Weining; FM, fresh matter; DM, dry matter; ND, not detected; “–”, default. D, ensiling days; T, treatment; D × T, interaction of ensiling days and treatment; SEM, standard error of means; Means with different letters in the same row (a–f) or column (A–C) differ (*P* < 0.05), ***P < 0.001.

### The Fermentation Property of Whole-Plant Maize During Ensiling

Table 2 illustrates the fermentation quality of whole-plant maize silage in different regions. The pH value of each group decreased significantly on the second day of fermentation (*P* < 0.05), all of which were below 4.2. Moreover, the highest and lowest pH appeared in the G group and the W group on day 45 (*P* < 0.05), respectively. The LA content of each group increased significantly during fermentation, and the highest LA content was found in the W group on day 45 (*P* < 0.05). The contents of AA and PA in each group gradually increased with prolonged ensiling, and the contents in the Z group were lower than those in the other groups on day 45 (*P* < 0.05). The contents of BA were not detected in all silages. The contents of AN/TN in all groups were dramatically increased (*P* < 0.05) during fermentation, within lower than 5%. Moreover, pH, AA, LA, PA, and AN/TN were interactively influenced by treatment and ensiling days (*p* < 0.001).

**TABLE 2:** Fermentation quality of whole-plant maize silage in different regions.

**Item**	**Treatment**	**Ensiling days**						**SEM**	***P*-value**		
		**0**	**2**	**5**	**10**	**20**	**45**		**D**	**T**	**D × T**
pH	Z	5.60Aa	3.76Cc	3.72Bc	3.67Bd	3.62Ce	3.80Bb	0.015	***	***	***
	G	5.59Aa	4.03Ab	3.87Ac	3.83Ac	3.78Ad	3.88Ac				
	W	5.56Aa	3.91Bb	3.73Bc	3.67Bd	3.66Bd	3.52Ce				
Lactic acid (g/kg DM)	Z	ND	31.5Ad	33.3Ac	34.4Ab	36.0Aa	29.2Be	0.179	***	***	***
	G	ND	22.5Ce	26.0Bc	27.7Bb	30.2Ca	23.5Cd				
	W	ND	24.9Bd	33.2Ac	34.3Ab	34.8Bb	36.1Aa				
Acetic acid (g/kg DM)	Z	ND	1.6Ae	2.4Ad	2.7Ac	3.4Ab	4.5Ca	0.067	***	***	***
	G	ND	1.3Be	1.9Bd	2.3Bc	2.9Bb	5.5Aa				
	W	ND	1.7Ad	2.4Ac	2.6Ac	3.7Ab	4.9Ba				
Propionic acid (g/kg DM)	Z	ND	0.8Cd	1.1Bc	1.4Bb	1.6Bb	1.9Ba	0.058	***	***	***
	G	ND	1.1Bd	1.9Ac	2.1Ac	2.3Ab	2.7Aa				
	W	ND	1.4Ae	1.7Ad	1.9Ac	2.2Ab	2.5Aa				
Butyric acid (g/kg DM)	Z	ND	ND	ND	ND	ND	ND	—	—	—	—
	G	ND	ND	ND	ND	ND	ND				
	W	ND	ND	ND	ND	ND	ND				
Ammonia-N (g/kg total N)	Z	15.9Be	19.5Bd	23.0Bc	23.7Bc	27.0Bb	34.4Ba	0.393	***	***	***
	G	15.5Bf	22.5Ae	25.1Ad	30.9Ac	34.8Ab	41.7Aa				
	W	20.1Ae	23.0Ad	23.9Bcd	24.8Bc	26.5Bb	31.9Ca				

Z, Ziyun; G, Guanling; W, Weining; FM, fresh matter; DM, dry matter; ND, not detected; “–”, default. D, ensiling days; T, treatment; D × T, interaction of ensiling days and treatment; SEM, standard error of means; Means with different letters in the same row (a–f) or column (A–C) differ (*P* < 0.05), ***P < 0.001.

### Microbial Diversity of Whole-Plant Maize During Ensiling

**FIGURE 2:**
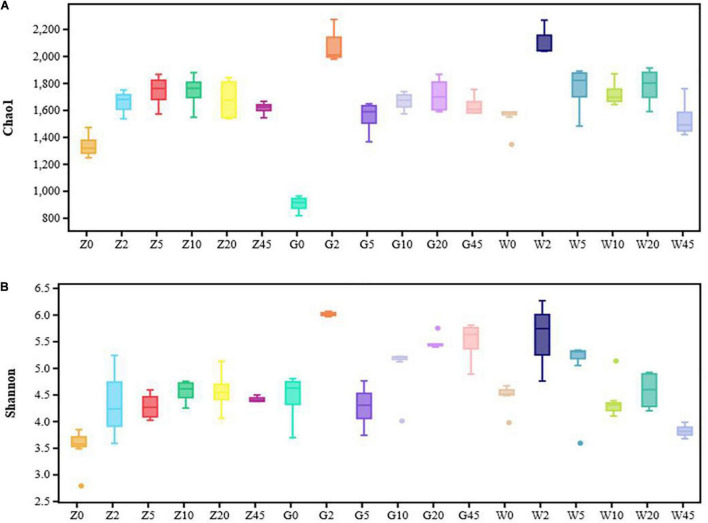
Community diversity and richness of the silages for every period in the area of Ziyun, Guanling, and Weining: **(A)** Chaol index of silage samples for every period (0, 2, 5, l0, 20, and 45 days); **(B)** Shannon index of silage samples for every period (0, 2, 5, l0, 20, and 45 days). Solid discrete points means outlier.

The bacterial alpha diversities of the silages were evaluated by the Chao1 and Shannon indexes (Figures 2A,B). The bacterial diversity increased with the extension of fermentation time in the Z group. However, the bacterial diversity of silages in the G group and the W group was richer after 2 days of fermentation.

**FIGURE 3:**
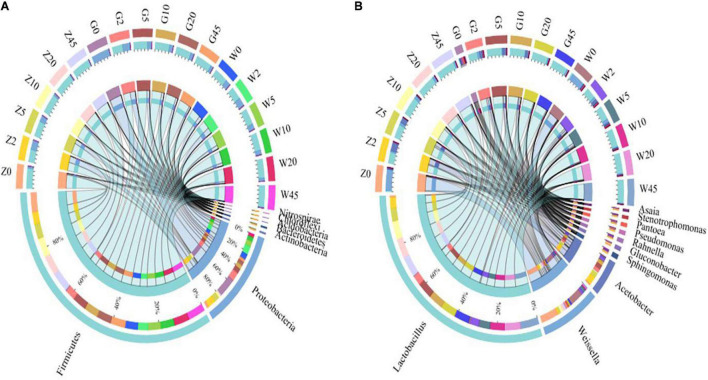
Circos map of bacterial communities at the phylum **(A)** and genus levels **(B)** for whole-plant maize silage.

**FIGURE 4:**
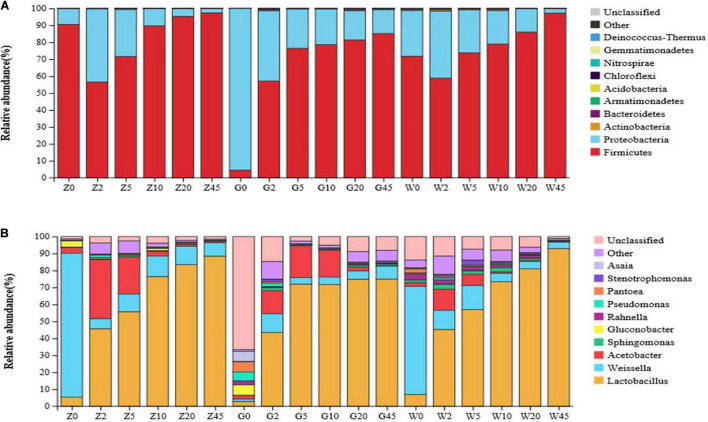
The relative abundance of bacteria community proportions at the phylum **(A)** and genus **(B)** level across the treatments (as a percentage of the total sequence).

The relative abundances of bacterial communities at the phylum and genus levels were presented in Figures 3, 4. As seen in Figure 3, Proteobacteria and Firmicutes were the top two phyla during the process of ensiling in the three regions. *Lactobacillus*, *Weissella*, and *Acetobacter* were the most dominant genera in all maize silages. Figure 4A shows the abundance of Proteobacteria was the most abundant phylum in the G group, exceeding 95% in raw materials. The dominant bacteria on the surface of maize in the Z and W groups were Firmicutes, with relative abundances of 90.55 and 71.70%, respectively, and Proteobacteria had relative abundances of 9.22 and 27.04%, respectively. At the genus level, *Weissella* and *Lactobacillus* were found in large amounts in the Z and W groups, while no obvious dominant bacteria were found in the G group of raw materials.

All the groups were dominated by Firmicutes and Proteobacteria during ensiling at the phylum level. The Proteobacteria abundance decreased while that of Firmicutes increased rapidly and became the dominant phylum with increasing fermentation time. At the genus level, the most dominant bacterial genera were *Lactobacillus* and *Weissella* in the samples during the ensiling process. After ensiling, *Lactobacillus* was the dominant microbial genus, and its relative abundance exceeded 70%. The *Lactobacillus* abundance significantly increased with the extension of fermentation time, while those of *Weissella* and *Acinetobacter* significantly decreased or disappeared in the subsequent period. The relative abundance of *Acetobacter* in each group decreased with the process of ensiling. By 45 days, the relative abundance of *Acetobacter* in each group decreased to below 1%. In addition, *Pantoea* and *Stenotrophomonas* existed in the whole corn silage period of all three regions.

### Correlation Analysis Between Bacterial Communities and Environmental Factors

**FIGURE 5:**
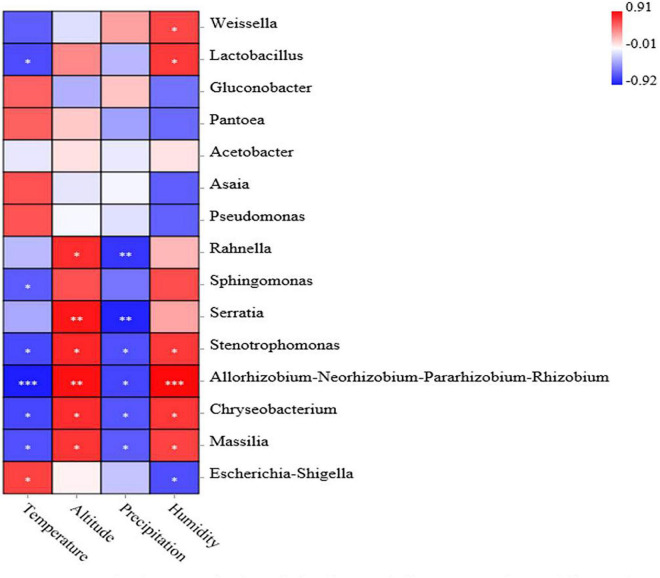
Correlation analysis of the bacterial community with environmental factors. Positive correlations are shown in red, and negative correlations are shown in blue. ‘^∗^, ^∗∗^, and ^∗∗∗^’ represents *P* < 0.05, *P* < 0.01, and *P* < 0.001, respectively.

The association analysis between bacterial abundance and environmental factors is shown in Figure 5. Negative correlations were observed between the average temperature and the relative abundance of the *Lactobacillus* (–0.73), *Sphingomonas* (–0.67), *Stenotrophomonas* (–0.74), *Allorhizobium-Neorhizobium-Pararhizobium-Rhizobium* (–0.92), *Chryseobacterium* (–0.75), and *Massilia* (–0.71). Altitude was associated with increased abundance of *Rahnella, Stenotrophomonas*, *Chryseobacterium*, *Massilia* (*p* < 0.05), *Serratia*, and *Allorhizobium-Neorhizobium-Pararhizobium-Rhizobium* (*p* < 0.01). The level of precipitation was associated with decreased abundance of *Rahnella*, *Serratia* (*p* < 0.01) and *Stenotrophomonas*, *Allorhizobium-Neorhizobium-Pararhizobium-Rhizobium*, *Chryseobacterium* and *Massilia* (*p* < 0.05). Humidity was associated with increased abundance of *Weissella*, *Lactobacillus*, *Stenotrophomonas*, *Chryseobacterium*, *Massilia* (*p* < 0.05), and *Allorhizobium-Neorhizobium-Pararhizobium-Rhizobium* (*p* < 0.001), while it was associated with decreased abundance of *Escherichia–Shigella* (*p* < 0.05).

### Correlation Analysis of Bacterial Community With Fermentation Products

**FIGURE 6:**
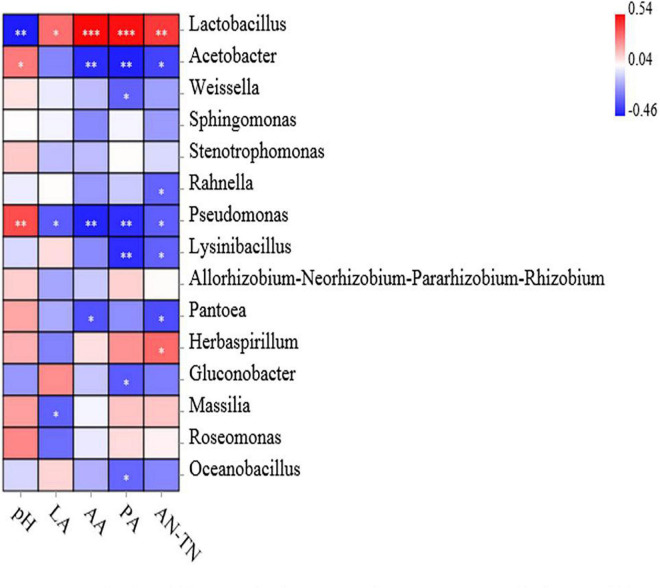
Relationships of fermentation characteristics with silage bacterial community at the genus level. Positive correlations are shown in red, and negative correlations are shown in blue. ‘^∗^, ^∗∗^, and ^∗∗∗^’ represent *P* < 0.05, *P* < 0.01, and *P* < 0.001, respectively.

The association analysis between bacterial abundance and fermentation indexes is shown in Figure 6. Specifically, the pH value was associated with decreased abundance of *Lactobacillus* (*p* < 0.01). The LA concentration was associated with increased abundance of *Lactobacillus* (*p* < 0.05), while it was negatively correlated with that of *Pseudomonas* and *Massilia* (*p* < 0.05). The contents of AA and PA were associated with increased abundance of *Lactobacillus* (*p* < 0.001), while AA concentration was negatively correlated with the abundance of *Acetobacter* and *Pseudomonas* (*p* < 0.01) and *Pantonea* (*p* < 0.05), PA concentration was associated with decreased abundance of *Acetobacter*, *Pseudomonas* and *Lysinibacillus* (*p* < 0.01) and *Weissella*, *Gluconobacter*, and *Oceanobacillus* (*p* < 0.05). Finally, the positive correlations have been observed between AN/TN concentrations and *Lactobacillus* (*p* < 0.01) and *Herbaspirillum* (*p* < 0.05), while AN/TN concentrations was associated with decreased abundance of *Acetobacter*, *Rahnella*, *Pseudomonas*, *Lysinibacillus*, and *Pantoea* (*p* < 0.05).

## Discussion

### Nutritional Characteristics of Whole-Plant Maize During Ensiling

The expected DM content for good silage is 30∼35% (Guyader et al., 2018). The DM content in each group decreased with the extension of fermentation time, which was mainly due to the WSC being consumed by LAB and other microorganisms for fermentation (Hu et al., 2009). The different CP contents of raw materials may be related to cultivation and fertilization (Miao et al., 2006). CP is one of the main nutritional components of silage, and the degradation of protein will affect the nutritional value of pastures. The lost nitrogen can only meet the nutritional needs of livestock by supplementing protein feed in the diet, thus increasing the breeding cost (Bilal, 2009). In this study, the CP content of the Z and G groups increased on the 10th and 20th days, respectively, while that of the W group increased on the 5th and 10th days of fermentation, which may be due to the decrease in DM content (Zhang et al., 2018). In addition, when the pH was low, some bacteria composed of protein could not grow and reproduce in the fermentation process and became a part of the feed, which also increased the content of CP (Bilal, 2009). On the other hand, the loss in CP was relatively small due to good fermentation.

The WSC content decreased significantly throughout the fermentation process, and the loss rate of each group reached 70%. As the substrate of silage fermentation, the WSC content will be decomposed by LAB to produce LA (Moselhy et al., 2015). The content of ADF and NDF affects the chewing time of ruminants and indirectly affects feed digestibility in domestic animals (Jang et al., 2017). In the present study, the contents of NDF and ADF in each group decreased with increasing fermentation time, which indicated that silage fermentation had a certain degradation effect on the fiber components of whole-plant maize, thus improving the digestibility of silage.

### The Fermentation Property of Whole-Plant Maize During Ensiling

The pH value of silage is an important index to evaluate the success of silage, and well-fermented silage should have a pH of 3.8∼4.2 (Kung et al., 2018). The low pH value ensures that harmful bacteria are inhibited and finally contributes to the good fermentation of whole-plant maize (Keshri et al., 2018). The pH was less than 4.0 and reached the lowest point at 20 days in all the silages except for that in the W group, which agreed with the results of a previous study (Sun et al., 2021). According to the fermentation mode, LA fermentation can be divided into homofermentative and heterofermentative types (Guo et al., 2018). Homofermentation mainly produces LA, whereas heterofermentation also produces AA, ethanol and CO_2_ in addition to LA (Pahlow et al., 2003). In this study, LAB grew rapidly and produced enough LA in each group, which inhibited the growth and reproduction of harmful microorganisms. With increasing LA content, homofermentative LAB are inhibited, while heterofermentative LAB begin to dominate due to the stronger tolerance to AA and pH value, and fermentation gradually changes from homofermentative LAB fermentation to heterofermentative LAB fermentation (Shao et al., 2002). The AA content of each group gradually increased with increasing fermentation time. In addition, when the silage is fermented to a certain extent and the pH is low, the fermentation of LAB will also be inhibited. At the same time, some anaerobic microorganisms that may exist in silage begin to decompose LA and produce other organic acids, such as AA and PA, which leads to a decrease in LA content (Shao et al., 2002). In this study, with the extension of fermentation time, the LA content decreased and the AA and PA contents increased gradually in the G group, which indicated that the type of fermentation was heterofermentative. The contents of LA and AA were relatively high, and the ammonia nitrogen content was within a limited range. These indexes showed the excellent fermentation quality of all the silages. BA is a product produced by decomposing protein, glucose and LA by spoilage bacteria and BA bacteria, respectively (Addah et al., 2012). It has been reported that BA content > 5 g/kg DM affects the palatability of feed and reduces the feed intake by livestock (Muck, 2010). During the whole fermentation process, BA was not detected in any group.

During the ensiling process, the ammonia nitrogen is mainly produced by the degradation of protein by plant enzymes and the utilization of protein and amino acids by microbial decomposition (Kung et al., 2018). In general, the ammonia-N content is recommended to be less than 5% for maize silage (Zhang et al., 2016). The high ammonia-N content indicates that proteolysis occurred to a deep extent during ensiling (Mu et al., 2021). In the present study, the content of AN/TN in each group was lower than 5%, indicating that harmful bacteria were effectively inhibited.

### Microbial Diversity of Whole-Plant Maize During Ensiling

The microbe numbers and species composition are varied with different silage materials or ensiling processes (Khota et al., 2016). Various epiphytic bacterial communities in raw materials might originate from unique growth environments (McGarvey et al., 2013). Gharechahi et al. (2017) reported a special bacterial community in whole-plant corn from different geographical locations. In this study, the differences at the species level in the epiphytic microorganisms of the Whole-plant fresh samples from the three areas were observed. The sampling locations in three areas belong to a subtropical monsoon climate and are close to each other. The phylum-level bacteria mainly attached to the surface of silage maize in the three regions were Firmicutes and Proteobacteria. Additionally, the main LAB genus in fresh forages was *Weissella*. This indicated that *Weissella* might play a major role in the early stage of fermentation (Sun et al., 2021). This genus is a selective anaerobic bacterium, which converts water-soluble sugars into LA and AA in the early stage of silage fermentation (Cai et al., 1998). In the current study, *Weissella* with low abundance was detected in silages during the ensiling process (Figure 4), which might lead to relatively high AA content in all silages (Table 2).

The nature of silage is due to complex microbial succession (Yang et al., 2019), in which bacteria play a key role (Li P. et al., 2019). In this study, the abundance of Firmicutes increased rapidly and became the dominant phylum during ensiling. This observation might be explained by the fact that the growth of Firmicutes is related to the low pH and anaerobic conditions formed during ensiling (Wang et al., 2019; Zhang et al., 2019). Although *Lactobacillus* was usually not the dominant bacteria in fresh corn of the Z group, it began to dominate at 2 days of ensiling. This observation suggested that the LAB count in the raw materials is enough to ensure the fermentation quality of the final silage. *Lactobacillus*, *Lactococcus, Weissella*, and *Enterococcus* are common LA-producing bacteria in silage, and their abundance changes are closely related to the quality of silage (Ni et al., 2018). Generally, LA-producing cocci are the dominant LAB. These initiate LA fermentation during the early stages of ensiling, whereby *Lactobacillus* will grow rapidly into the dominant bacteria (Liu et al., 2019). This result suggested that *Lactobacillus* was an important genus for silage fermentation during ensiling. Many studies have shown the dominance of *Lactobacillus* in ensiled corn silages (Li and Nishino, 2011; Ogunade et al., 2017) due to its desirable functions during ensiling (Li et al., 2017; Liu et al., 2019). The study showed that *Lactobacillus* and *Weissella* were the only LAB with high abundance in the whole-plant maize silage process in different regions, which was consistent with the research of Ogunade et al. (2017), and the reason for the difference with the research of Tohno et al. (2013) and Ni et al. (2017b) may be related to the research materials and locations.

*Gluconobacter* and *Acetobacter* were found in minor abundance during the early stages of ensilage. *Acetobacter* is a kind of AA-producing and nitrogen-fixing bacteria (Kumiko et al., 2001) and may lead to the decline in pH at the early stage of the ensiling process. Other bacteria, such as *Pantoea*, *Rahnella*, and *Sphingomonas*, were evenly distributed during the ensiling process in lower amounts are consistent with previous studies (Gharechahi et al., 2017; Ni et al., 2017a), and some of them are undesirable in silage (McDonald et al., 1991). Muck (2010) reported that *Bacillus*, *Paenibacillus*, *Enterobacter*, *Enterococcus*, and *Clostridium* are the main microorganisms that decompose proteins into ammonia nitrogen and cause protein loss. None of these bacteria were found in the top 10 bacteria regarding relative abundance, which was consistent with the previous results of low AN/TN contents in the present study.

### Correlation Analysis Between Bacterial Communities and Environmental Factors

There are many factors (moisture, WSC, regional factors) that affect the microbiome and influence the fermentation quality of silage (McEniry et al., 2010; Bernardes et al., 2018). Usually, regional factors include temperature, humidity, precipitation, longitude, latitude, and altitude. Previous reports showed that high temperatures and rainfall had detrimental effects on the fermentation process and silage quality (Kim and Adesogan, 2006). In addition, high temperature affected the transformation of microorganisms in corn silage from a homofermentative to a heterofermentative LAB community, which had been previously reported (Guan et al., 2020). Guan et al. (2018) reported that *Weissella*, *Pseudomonas*, and *Lactobacillus* were the main epiphytic bacteria of corn silage in high-temperature and high-humidity areas. Moreover, Zi et al. (2021b) reported that temperature, humidity, and precipitation affected the fermentation quality of silage through the changes in microbiomes. Epiphytic bacterial communities are highly related to climate (Bernardes et al., 2018). In this study, altitude was positively correlated with the abundance of *Stenotrophomonas*, *Chryseobacterium*, and *Massilia*, while precipitation *w*as negatively correlated with these bacteria. This indicates that precipitation was the factor affecting the epiphytic bacteria of the silage material. The *Lactobacillus* abundance in fresh maize did not change regularly with increasing altitude, and the reason for the difference may be related to the temperature and precipitation in the three regions. The correlation analysis (Figure 5) showed that the relative abundance of *Lactobacillus* was negatively correlated with temperature. This also explains why the temperature of the G group was the highest (21.35°C), while the relative abundance of attached *Lactobacillus* was the lowest (2.75%). The temperature of the W group was the lowest (17.32°C), while the relative abundance of attached *Lactobacillus* was the highest (63.77%). Altitude and precipitation influence some microorganisms in silage, but they do not relate to the main bacteria in silage, such as *Lactobacillus* and *Weissella*. This shows that when silage enters a completely anaerobic environment, the influence of climate factors on the main microorganisms will become smaller. Cai et al. (1999) reported that a certain amount of LAB in forage will have a great impact on silage fermentation. In this study, *Lactobacillus* and *Weissella* were correlated with temperature and humidity, and the most dominant bacterial genera were *Lactobacillus* and *Weissella* in the samples during the ensiling process. This result indicated that the main factors affecting the microbial diversity of silages were humidity and average temperature. Environmental factors affect the community of epiphytic bacteria on raw materials, which have a greater impact on the initial stage of silage fermentation (Zi et al., 2021b). We speculate that the influence of environmental factors on the microbial community will decrease during the silage fermentation process, thus reducing its influence on silage quality.

### Correlation Analysis of Bacterial Community With Fermentation Products

Microorganisms affect silage quality through a series of metabolites. For example, *Lactobacillus* species mainly affect LA production (Guan et al., 2018). In the present study, The *Lactobacillus* was positively correlated with the LA content, which dominated the bacterial community in the fermentation process and had a negative correlation with pH in all silages. This result was consistent with the report of Sun et al. (2021). *Pseudomonas* is an undesirable bacterium that can survive in an anaerobic environment, and its production of biogenic amines leads to the decrease in protein content (Roberson and Firestone, 1992; Dunière et al., 2013). The AN/TN content was negatively correlated with the abundance of *Pseudomonas*, indicating that the existence of *Pseudomonas* may contribute to the preservation of protein (Ogunade et al., 2018). Other studies have confirmed these findings, indicating that the fermentation characteristics are highly correlated with the microflora of silage and affect the overall fermentation quality (Ren et al., 2019; Yang et al., 2019).

## Conclusion

All of the whole-plant maize silages had satisfactory fermentation quality. The bacterial community of fresh raw material was mainly composed of *Weissella* and *Proteobacteria*. Although the bacterial community varied during ensiling, *Lactobacillus* dominated the ensiling process. *Lactobacillus* had a negative correlation with pH in all silages and grew well under low pH conditions, produced LA during ensiling, and effectively influenced fermentation quality. Altitude and precipitation influenced some specific microorganisms in silage, but they did not affect the main bacteria in silages. The humidity and average temperature significantly influenced the abundances of *Lactobacillus* and *Weissella* of fresh whole-plant maize and had a greater impact on the whole fermentation process.

## Data Availability Statement

The original contributions presented in the study are included in the article/supplementary material, further inquiries can be directed to the corresponding author/s.

## Author Contributions

JH and CC contributed to conception and design of the study. LL, SD, and CW performed the statistical analysis. YH wrote the manuscript and interpretation of data. All the authors read and contributed to the manuscript.

## Conflict of Interest

The authors declare that the research was conducted in the absence of any commercial or financial relationships that could be construed as a potential conflict of interest.

## Publisher’s Note

All claims expressed in this article are solely those of the authors and do not necessarily represent those of their affiliated organizations, or those of the publisher, the editors and the reviewers. Any product that may be evaluated in this article, or claim that may be made by its manufacturer, is not guaranteed or endorsed by the publisher.

## References

[B1] Addah W. Baah J. Okine E. K. McAllister T. A. (2012). A third-generation esterase inoculant alters fermentation pattern and improves aerobic stability of barley silage and the efficiency of body weight gain of growing feedlot cattle. *J. Anim. Sci.* 90 1541–1552. 10.2527/jas.2011-4085 22147468

[B2] AOAC (2000). *Official Methods of Analysis*, 17th Edn. Washington, DC: Association of Official Analytical Chemists.

[B3] Ashbell G. Weinberg Z. G. Hen Y. Filya I. (2002). The effects of temperature on the aerobic stability of wheat and corn silages. *J. Ind. Microb. Biotechnol*. 28 261–263. 10.1038/SJ/JIM/7000237 11986929

[B4] Bernardes T. F. Daniel J. L. P. Adesogan A. T. McAllister T. A. Drouin P. Nussio L. G. (2018). Silage review: unique challenges of silages made in hot and cold regions. *J. Dairy Sci.* 101 4001–4019. 10.3168/jds.2017-13703 29685274

[B5] Bilal M. Q. (2009). Effect of molasses and com silage addves on the characteristics of mott dwarf elephant grass silage at different fermentation periods. *Pakistan Vet. J.* 29 395–396.

[B6] Cai Y. M. Kumai S. Ogawa M. Benno Y. Nakase T. (1999). Characterization and identification of *Pediococcus* species isolated from forage crops and their application for silage preparation. *Appl. Environ. Microbiol*. 65 2901–2906. 10.1128/AEM.65.7.2901-2906.1999 10388681 PMC91434

[B7] Cai Y. Benno Y. Ogawa M. Ohmomo S. Kumai S. Nakase T. (1998). Influence of *Lactobacillus* spp. from an inoculant and of weissella and *Leuconostoc* spp. from forage crops on silage fermentation. *Appl. Environ. Microbiol*. 64 2982–2987. 10.1128/AEM.64.8.2982-2987.1998 9687461 PMC106803

[B8] Callahan B. J. Mcmurdie P. J. Rosen M. J. Han A. W. Johnson A. J. A. Holmes S. P. (2016). DADA2: high-resolution sample inference from illumine amplicon data. *Nat. Methods* 13 581–583. 10.1038/nmeth.3869 27214047 PMC4927377

[B9] Dunière L. Sindou J. Chaucheyras-Durand F. Chevallier I. Thévenot Sergentet D. (2013). Silage processing and strategies to prevent persistence of undesirable microorganisms. *Anim. Feed Sci. Technol*. 182 1–15. 10.1016/j.anifeedsci.2013.04.006

[B10] Gharechahi J. Kharazian Z. A. Sarikhan S. Jouzani G. S. Aghdasi M. Salekdeh G. H. (2017). The dynamics of the bacterial communities developed in maize silage. *Microb. Biotechnol*. 10 1663–1676. 10.1111/1751-7915.12751 28696065 PMC5658587

[B11] Guan H. Shuai Y. Yan Y. Ran Q. Wang X. Li D. (2020). Microbial community and fermentation dynamics of corn silage prepared with heat-resistant lactic acid bacteria in a hot environment. *Microorganisms* 8:719. 10.3390/microorganisms8050719 32408707 PMC7285033

[B12] Guan H. Yan Y. Li X. Li X. Shuai Y. Feng G. (2018). Microbial communities and natural fermentation of corn silages prepared with farm bunker-silo in Southwest China. *Bioresour. Technol.* 265 282–290. 29908496 10.1016/j.biortech.2018.06.018

[B13] Guo X. S. Ke W. C. Ding W. R. Ding L. M. Xu D. M. Wang W. W. (2018). Profiling of metabolome and bacterial community dynamics in ensiled *Medicago sativa* inoculated without or with *Lactobacillus plantarum* or *Lactobacillus buchneri*. *Sci. Rep*. 8 357–366. 10.1038/s41598-017-18348-0 29321642 PMC5762819

[B14] Guyader J. Baron V. Beauchemin K. (2018). Corn forage yield and quality for silage in short growing season areas of the Canadian prairies. *Agronomy* 8 164–176. 10.3390/agronomy8090164

[B15] Hu W. Schmidt R. J. Mcdonell E. E. Klingerman C. M. Kung L. Jr. (2009). The effect of *Lactobacillus buchneri* 40788 or *Lactobacillus plantarum* MTD-1 on the fermentation and aerobic stability of corn silages ensiled at two dry matter contents. *J. Dairy Sci*. 92 3907–3914. 10.3168/jds.2008-1788 19620673

[B16] Hu Z. Chang J. Yu J. Li S. Niu H. (2018). Diversity of bacterial community during ensiling and subsequent exposure to air in whole-plant maize silage. *Asian Australas. J. Anim. Sci*. 31 1464–1473. 10.5713/ajas.17.0860 29747496 PMC6127572

[B17] Jang S. Y. Kim E. K. Park J. H. Tang Y. J. M. Ding Y. L. Kim W. H. (2017). Effects of physically effective neutral detergent fiber content on dry matter intake, digestibility, and chewing activity in Korean native goats (*Capra hircus* coreanae) fed with total mixed ration. *Asian Australas. J. Anim. Sci*. 30 1405–1409. 10.5713/ajas.16.0868 28423870 PMC5582324

[B18] Keshri J. Chen Y. Pinto R. Kroupitski Y. Weinberg Z. G. Sela S. (2018). Microbiome dynamics during ensiling of corn with and without *Lactobacillus plantarum* inoculant. *Appl. Microbiol. Biot*. 102 4025–4037. 10.1007/s00253-018-8903-y 29536147

[B19] Khan N. A. Yu P. Ali M. Cone J. W. Hendriks W. H. (2015). Nutritive value of maize silage in relation to dairy cow performance and milk quality. *J. Sci. Food Agr*. 95 238–252. 10.1002/jsfa.6703 24752455

[B20] Khota W. Pholsen S. Higgs D. Cai Y. (2016). Natural lactic acid bacteria population of tropical grasses and their fermentation factor analysis of silage prepared with cellulase and inoculant. *J. Dairy Sci*. 99 9768–9781. 10.3168/jds.2016-11180 27743669

[B21] Kim S. C. Adesogan A. T. (2006). Influence of ensiling temperature, simulated rainfall, and delayed sealing on fermentation characteristics and aerobic stability of corn silage. *J. Dairy Sci.* 89 3122–3132. 10.3168/jds.s0022-0302(06)72586-3 16840629

[B22] Kumiko N. Taniguchi M. Ujike S. Ishihara N. Mori H. Ono H. (2001). Characterization of *acetic acid bacteria* in traditional acetic acid fermentation of rice vinegar (komesu) and unpolished rice vinegar (kurosu) produced in Japan. *Appl. Environ. Microbiol*. 67 986–990. 10.1128/AEM.67.2.986-990.2001 11157275 PMC92679

[B23] Kung L. Jr. Shaver R. D. Grant R. J. Schmidt R. J. (2018). Silage review: interpretation of chemical, microbial, and organoleptic components of silages. *J. Dairy Sci.* 101 4020–4033. 10.3168/jds.2017-13909 29685275

[B24] Li L. Yuan Z. Sun Y. Kong X. Dong P. Zhang J. (2017). A reused method for molasses-processed wastewater: effect on silage quality and anaerobic digestion performance of *Pennisetum purpereum*. *Bioresour. Technol.* 241 1003–1011. 10.1016/j.biortech.2017.04.117 28637158

[B25] Li M. Zi X. Tang J. Zhou H. Cai Y. (2019). Silage fermentation, chemical composition and ruminal degradation of king grass, cassava foliage and their mixture. *Grassland Sci.* 64 210–215. 10.1111/grs.12235

[B26] Li P. Zhang Y. Gou W. Cheng Q. Bai S. Cai Y. (2019). Silage fermentation and bacterial community of bur clover, annual ryegrass and their mixtures prepared with microbial inoculant and chemical additive. *Anim. Feed Sci. Technol.* 247 285–293. 10.1016/j.anifeedsci.2018.11.009

[B27] Li X. M. Chen F. Wang X. K. Sun L. Guo L. N. Xiong Y. (2021). Impacts of low temperature and ensiling period on the bacterial community of oat silage by SMRT. *Microorganisms* 9:274. 10.3390/MICROORGANISMS9020274 33525587 PMC7910925

[B28] Li Y. Nishino N. (2011). Monitoring the bacterial community of maize silage stored in a bunker silo inoculated with *Enterococcus faecium*. *Lactobacillus plantarum* and *Lactobacillus buchneri*. *J. Appl. Microbiol.* 110 1561–1570. 10.1111/j.1365-2672.2011.05010.x 21447012

[B29] Lin C. Bolsen K. K. Brent B. E. Daniel Y. C. F. (1992). Epiphytic lactic acid bacteria succession during the pre-ensiling and ensiling periods of alfalfa and maize. *J. Appl. Microbio*. 73 375–387.

[B30] Liu B. Huan H. Gu H. Xu N. Shen Q. Ding C. (2019). Dynamics of a microbial community during ensiling and upon aerobic exposure inlactic acid bacteria inoculation-treated and untreated barley silages. *Bioresour. Technol.* 273 212–219. 10.1016/j.biortech.2018.10.041 30447622

[B31] Logue J. B. Stedmon C. A. Kellerman A. M. Nielsen N. J. Andersson A. F. (2016). Experimental insights into the importance of aquatic bacterial community composition to the degradation of dissolved organic matter. *ISME J.* 10 533–545. 10.1038/ismej.2015.131 26296065 PMC4817675

[B32] McDonald P. Henderson A. R. Heron S. J. E. (1991). *The Biochemistry of Silage. Version*, 2th Edn. Marlow: Chalcombe Publications.

[B33] McEniry J. O’Kiely P. Clipson N. Forristal P. Doyle E. (2010). Assessing the impact of various ensilage factors on the fermentation of grass silage using conventional culture and bacterial community analysis techniques. *J. Appl. Microbiol.* 108 1584–1593. 10.1111/j.1365-2672.2009.04557.x 19863691

[B34] McGarvey J. Franco R. Palumbo J. Hnasko R. Stanker L. Mitloehner F. (2013). Bacterial population dynamics during the ensiling of *Medicago sativa* (alfalfa) and subsequent exposure to air. *J. Appl. Microbiol.* 114 1661–1670. 10.1111/jam.12179 23521112

[B35] Miao Y. Mulla D. J. Robert P. C. Hernandez J. A. (2006). Within-field variation in corn yield and grain quality responses to nitrogen fertilization and hybrid selection. *Agron. J.* 98 129–140. 10.2134/agronj2005.0120

[B36] Moselhy M. A. Borba J. P. Borba A. E. S. (2015). Improving the nutritive value, in vitro digestibility and aerobic stability of *Hedychium gardnerianum* silage through application of additives at ensiling time. *Anim. Feed Sci. Technol.* 206 8–18. 10.1016/j.anifeedsci.2015.05.001

[B37] Mu L. Xie Z. Hu L. Chen G. Zhang Z. (2021). *Lactobacillus plantarum* and molasses alter dynamic chemical composition, microbial community, and aerobic stability of mixed (amaranth and rice straw) silage. *J. Sci. Food. Agr*. 101 5225–5235. 10.1002/JSFA.11171 33611793

[B38] Muck R. (2010). Silage microbiology and its control through additives. *Rev. Bras. Zootec.* 39 183–191. 10.1590/S1516-35982010001300021

[B39] Ni K. K. Wang F. F. Zhu B. G. Yang J. X. Zhou G. A. (2017b). Effects of lactic acid bacteria and molasses additives on the microbial community and fermentation quality of soybean silage. *Bioresour. Technol.* 238 706–715. 10.1016/j.biortech.2017.04.055 28501002

[B40] Ni K. K. Minh T. T. Tu T. T. (2017a). Comparative microbiota assessment of wilted Italian ryegrass, whole crop corn, and wilted alfalfa silage using denaturing gradient gel electrophoresis and next-generation sequencing. *Appl. Microbiol. Biotechnol.* 101 1385–1394. 10.1007/s00253-016-7900-2 27722778

[B41] Ni K. Zhao J. Zhu B. Su R. Pan Y. Ma J. (2018). Assessing the fermentation quality and microbial community of the mixed silage of forage soybean with crop corn or sorghum. *Bioresour. Technol.* 265 563–567. 10.1016/j.biortech.2018.05.097 29861298

[B42] Ogunade I. M. Jiang Y. Kim D. H. Cervantes A. A. P. Arriola K. G. Vyas D. (2017). Fate of *E. coli* O157:H7 and bacterial diversity in corn silage contaminated with the pathogen and treated with chemical or microbial additives. *J. Dairy Sci*. 100 1780–1794. 10.3168/jds.2016-11745 28041727

[B43] Ogunade I. M. Jiang Y. Pech Cervantes A. A. Kim D. H. Oliveira A. S. Vyas D. (2018). Bacterial diversity and composition of alfalfa silage as analyzed by Illumina MiSeq sequencing: effects of *Escherichia coli* O157:H7 and silage additives. *J. Dairy Sci.* 101 2048–2059. 10.3168/jds.2017-12876 29274960

[B44] Pahlow G. Muck R. E. Driehuis F. Oude Elferink S. J. W. H. Spoelstra S. F. (2003). “Microbiology of ensiling,” in *Silage Science and Technology*, eds Buxton D. R. Muck R. E. Harrison J. H. (Madison, WI: American Society of Agronomy), 31–93. 10.2134/agronmonogr42.c2

[B45] Pang H. Qin G. Tan Z. Li Z. Wang Y. Cai Y. (2011). Natural populations of lactic acid bacteria associated with silage fermentation as determined by phenotype, 16 S ribosomal RNA and recA gene analysis. *Syst. Appl. Microbiol.* 34 235–241. 10.1016/j.syapm.2010.10.003 21282025

[B46] Ren F. He R. Zhou X. Gu Q. Xia Z. Liang M. (2019). Dynamic changes in fermentation profiles and bacterial community composition during sugarcane top silage fermentation: a preliminary study. *Bioresour. Technol*. 285:121315. 10.1016/j.biortech.2019.121315 30965280

[B47] Roberson E. B. Firestone M. K. (1992). Relationship between desiccation and exopolysaccharide production in a soil *Pseudomonas* sp. *Appl. Environ. Microb.* 58 1284–1291. 10.1128/AEM.58.4.1284-1291.1992 16348695 PMC195588

[B48] Schloss P. D. Westcott S. L. Ryabin T. Hall J. R. Hartmann M. Hollister E. B. (2009). Introducing mothur: open-source, platform-independent, community-supported software for describing and comparing microbial communities. *Appl. Environ. Microbiol.* 75 7537–7541. 10.1128/aem.01541-09 19801464 PMC2786419

[B49] Sepehri A. Sarrafzadeh M. (2019). Activity enhancement of ammonia-oxidizing bacteria and nitrite-oxidizing bacteria in activated sludge process: metabolite reduction and CO2mitigation intensification process. *Appl. Water Sci.* 9:131. 10.1007/s13201-019-1017-6

[B50] Shao T. Ohba N. Shimojo M. Masuda Y. (2002). Dynamics of early fermentation of Italian ryegrass (*Lolium multiflorum* Lam.)silage. *Asian Aust. J. Anim. Sci.* 15 1606–1610.

[B51] Sun L. Bai C. Xu H. Na N. Jiang Y. Yin G. (2021). Succession of bacterial community during the initial aerobic, intense fermentation, and stable phases of whole-plant corn silages treated with lactic acid bacteria suspensions prepared from other silages. *Front. Microbiol*. 12:655095. 10.3389/FMICB.2021.655095 33841382 PMC8032959

[B52] Tohno M. Kitahara M. Irisawa T. Masuda T. Uegaki R. Ohkuma M. (2013). *Lactobacillus silagei* sp. nov. Isolated from orchardgrass silage. *Int. J. Syst. Evol. Microbiol.* 63 4613–4618. 10.1099/ijs.0.053124-0 23919960

[B53] Turula V. E. J. Gore T. Singh S. Arumugham R. G. (2010). Automation of the anthrone assay for carbohydrate concentration determinations. *Anal. Chem*. 82 1786–1792. 10.1021/ac902664x 20121220

[B54] Van Soest P. J. Robertson J. B. Lewis B. A. (1991). Methods for dietary fiber, neutral detergent fiber, and nonstarch polysaccharides in relation to animal nutrition. *J. Dairy Sci.* 74 3583–3597. 10.3168/jds.s0022-0302(91)78551-2 1660498

[B55] Vázquez-Baeza Y. Meg P. Antonio G. (2013). EMPeror: a tool for visualizing high-throughput microbial community data. *GigaScience* 2:16. 24280061 10.1186/2047-217X-2-16 PMC4076506

[B56] Wang Y. Chen X. Wang C. He L. Zhou W. Yang F. (2019). The bacterial community and fermentation quality of mulberry (*Morus alba*) leaf silage with or without *Lactobacillus casei* and sucrose. *Bioresour. Technol*. 293 122059. 10.1016/j.biortech.2019.122059 31476563

[B57] Xu D. Ding W. Ke W. Li F. Zhang P. Guo X. (2019). Modulation of metabolome and bacterial community in whole crop corn silage by inoculating homofermentative *Lactobacillus plantarum* and Heterofermentative *Lactobacillus buchneri*. *Front. Microbiol*. 9:3299. 10.3389/FMICB.2018.03299 30728817 PMC6352740

[B58] Yang L. Yuan X. Li J. Dong Z. Shao T. (2019). Dynamics of microbial community and fermentation quality during ensiling of sterile and nonsterile alfalfa with or without *Lactobacillus plantarum* inoculant. *Bioresour. Technol.* 275 280–287. 10.1016/j.biortech.2018.12.067 30594838

[B59] Yuan X. J. Li J. F. Dong Z. H. Shao T. (2019). The reconstitution mechanism of napier grass microiota during the ensiling of alfalfa and their contributions to fermentation quality of silage. *Bioresour. Technol*. 297:122391. 10.1016/j.biortech.2019.122391 31759854

[B60] Zhang L. Zhou X. Gu Q. Liang M. Mu S. Zhou B. (2019). Analysis of the correlation between bacteria and fungi in sugarcane tops silage prior to and after aerobic exposure. *Bioresour. Technol*. 291:121835. 31352166 10.1016/j.biortech.2019.121835

[B61] Zhang Q. Yu Z. Wang X. Tian J. (2018). Effects of inoculants and environmental temperature on fermentation quality and bacterial diversity of alfalfa silage. *Anim. Sci. J*. 89 1085–1092. 10.1111/asj.12961 29845704

[B62] Zhang Y. Yang J. Wang J. Zheng N. Li S. Zhao S. (2016). Progress assessment of chemical indicators of silage. *Chinese J. Anim. Husbandry*. 52 37–42.

[B63] Zhou Y. Drouin P. Lafreniere C. (2016). Effect of temperature (5-25°C) on epiphytic lactic acid bacteria populations and fermentation of whole-plant corn silage. *J. Appl. Microbiol*. 121 657–671. 10.1111/jam.13198 27271320

[B64] Zhu M. F. Xie Y. N. Li S. J. Chen X. W. Zhao Z. (2010). Technical measures for improving quality of silage maize in Southern area. *Anim. Husb. Feed Sci*. 31 31–34.

[B65] Zi X. Li M. Chen Y. Lv R. Zhou H. Tang J. (2021a). Effects of citric acid and *Lactobacillus plantarum* on silage quality and bacterial diversity of king grass silage. *Front. Microbiol*. 12:631096. 10.3389/fmicb.2021.631096 33717021 PMC7953137

[B66] Zi X. Li M. Yu D. Tang J. Zhou H. Chen Y. (2021b). Natural fermentation quality and bacterial community of 12 pennisetum sinese varieties in Southern China. *Front. Microbiol.* 12:627820. 10.3389/fmicb.2021.627820 33995292 PMC8116707

